# Surgical Excision of Plantar Wart

**DOI:** 10.1111/srt.70327

**Published:** 2026-04-16

**Authors:** María José Chiva Miralles

**Affiliations:** ^1^ Department of Nursing University of Valencia València Valencia Spain

**Keywords:** excision, plantar warts, surgery, treatment

## Abstract

**B&G:**

Plantar warts are common reasons for consultation and present multiple treatments as well as varying results.

**Methods:**

This clinical case demonstrates the step‐by‐step process for surgical removal of plantar warts.

**Results:**

Healing times are shorter than with conventional treatments.

**Conclusion:**

Surgical removal of plantar warts is a first‐line treatment.

## 1 Background

Plantar warts are lesions that appear solitary, deep, and painful. The annual incidence in the population is 14%, affecting both young and adult patients. Currently there is a wide variety of topical treatments, but none have been proven to be effective with all patients and they tend to recur. The objective of this article is the visualization and dissemination of the surgical procedure for the removal of the plantar wart; since it is a simple procedure. The lesion was surgically removed at the level of the pad of the first toe with excellent results and no recurrences.

Plantar warts are one of the most common skin diseases on the sole. Different authors state that the annual incidence in the population is 14% [1, 2, 3].

Plantar warts are lesions that appear solitary, deep, and painful. The description of a plantar wart is characterized by two clinical aspects, the first of which is that the lesion is covered by a circumscribed keratotic layer/plate called a keratotic ring, and the second identity characteristic of this lesion is that in the central area of the wart, bleeding or black spots appear. This manifestation is due to the presence of thrombosed capillaries in the wart stroma. These types of warts are capable of compressing the nerve endings due to their depth and causing pain during standing both statically and dynamically as a result of their location and endophytic growth. Its diameter under normal conditions ranges between 2 and 10 mm [3]. They are usually located in pressure points on the forefoot, under the metatarsal heads and toes, or on the heel. They are produced in most cases by HPV type 57, 27, although viral types 1, 2, and 65 are also common [4].

Currently, there's a wide variety of topical treatments, but none have proven to be effective with all patients and they tend to recur [3]. All the authors affirm that the treatment for warts must be effective, cheap, simple, and with minimal adverse effects. It should be noted that there're multiple studies on topical treatments, but the presence of studies on plantar wart surgery is almost non‐existent, only publications of clinical cases in which there's suspicion of malignancy [5] and/or under study sample without representative results [2]. Saipoor presents a study on a plastic surgery for plantar injuries but with long healing times [6]. Unlike the study by Mahrle and Alexandre, in which in addition to curettage of the lesion, they performed electrocoagulation of the cavity [7].

## 2 Methods

The objective of the author is the visualization and dissemination of the surgical procedure for the extirpation of the plantar wart to increase its use among the different health professionals who deal with the pathology, since experience gives us security.

Surgical removal of the lesion is a simple procedure, but it is an invasive procedure. Like any surgical process, it is performed under anesthesia; This will always be carried out under the criteria of the professional, performing the anesthetic block that best suits the needs of the patient, always considering the location of the lesion. For example, if the wart is located on the first toe, this will block troncal anesthesia. If the lesion is located on the heel, a tibial nerve block will be performed, with retromalleolar approach; since performing an infiltration directly on the sole of the foot, in addition to being very painful for the patient, is not recommended. Another relevant aspect in surgery is hemostasis. If the lesion is located in the toes, we can perform direct hemostasis, with the help of tourniquet rings, which are marketed with different diameters. If the lesion is in the area of the metatarsal heads, heel, or ALI, hemostasis is performed mechanically, applying pressure to both sides of the lesion throughout the intervention, preventing bleeding due to compression of the surrounding vessels. The fact of working in a bloodless field is for a better visualization of the tissues by the physician.

It should be noted that in none of the cases is cautery used in the cavity of the lesion, since this is an epithelial tumor and with the application of gauzes in the form of a plug, hemorrhage and coagulation are controlled in the post‐surgical process.

The necessary material to carry out the surgical excision is the following: scalpel handle number 3, scalpel blade number 15, Addson forceps with and without teeth, mosquito forceps, Martin and/or Volkman spoon, gauze, saline solution, and syringe.

**FIGURE 1:**
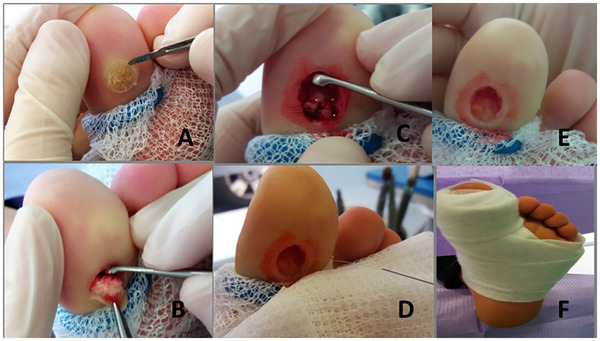
**Surgical procedure for excision of the plantar wart (A–F)**. (A) Perilesional incision with a scalpel, in a lesion on the 1st finger with application of a hemostatic tourniquet. (B) With the help of tweezers and spoon, the tumor is extracted. (C) Curettage of the cavity. (D) Washing with physiological saline under pressure. (E) surgical cavity after excision. (F) Post‐surgical bandage.

Surgical procedure (Figure 1): with the tip of the scalpel, a superficial incision is made completely skirting the perimeter of the wart, leaving a 2–3 mm safety margin (Figure 1A). Once this first incision has been made, which helps us to delimit the field of work, the spoon is introduced through it, making pressure and dragging movements (Figure 1B), introducing even deeper planes, in order to extract the entire tumor. At the same time, with the help of forceps, we are holding the verrucous tumor from one end and traction is performed so that complete exeresis can be performed with the spoon (Figure 1C). Once the lesion has been completely removed with the spoon, a disto‐proximal curettage of the cavity is performed to prevent contaminated cells from remaining (Figure 1D). Subsequently, washing with physiological saline (Figure 1C) and drying is carried out. Finally, gauze is applied to the cavity obtained (Figure 1E) with a hemostatic effect. We apply a semi‐compressive bandage (Figure 1F) that we remove after 48 h.

## 3 Results

As regards the excised lesion, it is referred to the pathological anatomy service for analysis and diagnostic confirmation. At 48 h after surgery, the bandage is removed, and daily cures with healing ointments to help epithelization begin. These cures can either be performed by the patient at home or go to the consultation to be performed by the professional. The cures consist of washing the area with physiological saline, drying and subsequent application of healing ointment, to allow closure by secondary intention and achieve complete epithelialization after approximately 2 weeks.

The criteria for surgical removal are determined by both the physician and the patient, after having provided various treatment options, provided that the lesion meets the following criteria: size between 2 and 10 mm, is a single lesion, and the clinical characteristics indicate that we are dealing with a plantar wart.

## 4 Conclusions

Surgical removal of plantar warts, with all the healing time, as long as the lesion is between 2 and 10 mm in size, has a healing time of approximately 2 weeks.

This healing time is shorter than that of many commonly used conventional treatments. Therefore, wart surgery should be considered as a first line of treatment.

## Consent

Informed written consent was obtained from the patient. The patient's identity remains confidential throughout the report.

## Conflicts of Interest

The author declares no conflicts of interest.

## Data Availability

This report is descriptive, based on clinical observations and narrative descriptions. As such, there are no data to be shared.

## References

[1] P. Gerlero and A. Hernández‐Martín , “Actualización sobre el Tratamiento de las Verrugas Vulgares en los Niños,” Actas Dermo‐Sifiliograficas 107 (2016): 445–450. 10.1016/j.ad.2016.04.010 27241712

[2] J. Hekmatjah , M. Farshchian , J. M. Grant‐Kels , and D. Mehregan , “The Status of Treatment for Plantar Warts in 2021: No Definitive Advancements in Decades for a Common Dermatology Disease,” Clinics in Dermatology 39 (2021): 688–694. 34809773 10.1016/j.clindermatol.2021.05.024

[3] A. Martínez Nova , R. Sánchez Rodríguez , B. Gómez Martín , E. Escamilla Martínez , and V. Cáceres Madrid , “Infeccciones Víricas y Mixtas Más Frecuentes en el Pie,” Revista Española de Podología 21, no. 6 (2010): 230–236.

[4] E. de Planell‐Mas , B. Martínez‐Garriga , A. J. Zalacaín , T. Vinuesa , and M. Viñas , “Human Papiloma Viruses Genotyping in Plantar Warts,” Journal of Medical Virology 89, no. 5 (2017): 902–907. 27736001 10.1002/jmv.24713

[5] D. K. Gordon , E. N. Ponder , B. Hudson Berrey , M. J. Kubik , and J. Sindone , “Verrucous Carcinoma of the Foot, Not Your Typical Plantar Wart: A Case Study,” Foot 22, no. 2 (2014): 94–98. 10.1016/j.foot.2014.03.009 24810296

[6] A. Saipoor , A. Maher , and L. Hogg , “A Retrospective Audit of Lesion Excision and Rotation Skin Flap for the Treatment of Intractable Plantar Keratosis,” Foot (Edinb) 34 (2018): 23–27. 29202430 10.1016/j.foot.2017.09.004

[7] G. Mahrle and W. Alexander , “Surgical Treatment of Recalcitrant Warts,” Journal of Dermatologic Surgery and Oncology 9, no. 6 (1983): 445–450. 6853811 10.1111/j.1524-4725.1983.tb00833.x

